# Data of plant diversity, spectral reflectance at specie level and satellite spectral variables from the largest dry forest nucleus in South America

**DOI:** 10.1016/j.dib.2019.104335

**Published:** 2019-07-29

**Authors:** Edna Samara e Silva Medeiros, Célia Cristina Clemente Machado, Josiclêda Domiciano Galvíncio, Magna Soelma Beserra de Moura, Helder Farias Pereira de Araujo

**Affiliations:** a Universidade Federal da Paraíba, Campus II, Centro de Ciências Agrárias, Cep: 58.397.000, Areia, PB, Brazil; b Universidade Estadual da Paraíba, Campus V, Centro de Ciências Biológicas e Sociais Aplicadas, Cep: 58071-160, Cristo Redentor - João Pessoa, PB, Brazil; c Universidade Federal de Pernambuco, Centro de Filosofia e Ciências Geográficas, Cep: 50.670901, Recife, PE, Brazil; d Empresa Brasileira de Pesquisa Agropecuária, Centro de Pesquisa Agropecuária do Trópico Semiárido, Cep: 56.302970, Petrolina, PE, Brazil

**Keywords:** Richness, Reflectance, Remote sensing

## Abstract

The use of satellite remote sensing makes it possible to acquire useful information about the environment, since it presents tools capable of assisting the practical search of information related to species richness. Here we present data on richness and Shannon index from phytosociological researches, vegetation indices and individual bands spectral reflectance from satellite images and leaf-level spectral reflectance from eight Caatinga species. For further interpretation of the data presented in this article, please see the research article “Predicting plant species richness with satellite images in the largest dry forest nucleus in South America” [1].

**Table:** Specifications Table

Subject area	Biology
More specific subject area	Remote Sensing
Type of data	Table and graph
How data was acquired	Plant diversity data (richness and Shannon index) were obtained through searches of phytosociological studies carried out in the study area. Spectral bands and vegetation indices were obtained with information from Landsat 5 and 8 satellites. The leaf spectral reflectance between 336 and 1045 nm with a resolution of 1 nm was measured using a spectroradiometer (model FieldSpec® HandHeld Pro) and it used a 1 and 10° HH FOV lens foreoptic with radiometric calibration.
Data format	Raw and analyzed
Experimental factors	Eight Vegetation indices (NDVI, EVI, LAI_MAC, LAI_GALV, NDWI, SAVI and SR) and six spectral bands (BLUE, GREEN, RED, NIR, SWIR1 and SWIR2)
Experimental features	Correlation of data of plant diversity and spectral variables
Data source location	Dry forest region, Brazil, between latitudes 2° 49′ 46″ S and 17° 10′ 57″ S and longitudes 35° 10′36″ W and 45° 26′14″ W.
Data accessibility	All data presented in this article.
Related research article	E. S. S. Medeiros, C. C. C. Machado, J. D. Galvíncio, M. S. B. Moura, H. F. P. Araújo. Predicting plant species richness with satellite images in the largest dry forest nucleus in South America . Journal of Arid Environments, 166, 2019, 0140–1963 [1]

**Table:** 

**Value of the data** •The spectral variables data can be used to associate with diversity and structural vegetation data in Caatinga •The leaf level near-infrared (NIR) spectral region was the best reflectance information to differentiate plant species •The data may be relevant for future researchers about biodiversity in the Caatinga, for example, for ecological modeling

## 1 Data

**Fig. 1:**
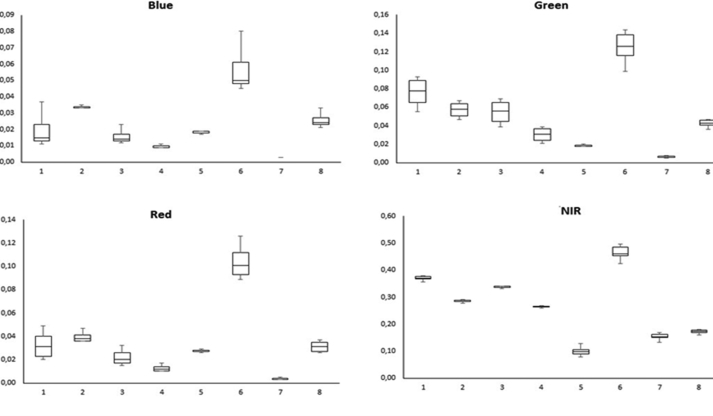
Leaf-level spectral reflectance in eight Caatinga plant species: 1-*Manihot glaziovii;*2- *Croton sonderianus;* 3-*Jatropha mollissima;* 4- *Croton conduplicatus;* 5- *Commiphora leptophloeos;* 6-*Bauhinia sp*.,7-*Capparis flexuosa L*., and 8-*Cereus jamacaru*. Spectral bands: Blue, Green, Red and Near-infrared (NIR).

In this report, we present richness and Shannon index data that were extracted from phytosociological researches carried out in Caatinga region, the largest dry forest nucleus in South America. The values of individual bands reflectance and vegetation indices from the same sites where phytosociological researches were carried are shown in Table 1. The foliar level reflectance from eight plant species varied among the Blue, Green, Red and NIR spectral regions. The near-infrared (NIR) is the one that presents the greatest power to differentiate eight Caatinga common species (Fig. 1).

**Table 1:** Raw data of plant species richness and Shannon index of sites in largest nucleus of dry forests in South America and respective vegetation indexes and individual spectral bands extracted from the satellite image information.

POINT		REFERENCE	R	S	NDVI	EVI	LAF MAC	LAF GALV	NDWI	SAVI	SR	DVI	BLUE	GREEN	RED	NIR	SWIR 1	SWIR 2
X	Y																	
−6.591388	−37.25083	Fabricante, J.R., 2007 [19]	25		0.1832475	0.1433715	0.27204725	0.217529	−0.144759	0.156281	1.449306	0.063341	0.1265785	0.125525	0.14175	0.20525	0.274	0.1765
−6.81	−36.96058	Fabricante, J.R., 2007 [19]	20		0.2392752	0.1938307	0.36794925	0.432878	−0.078144	0.2116372	1.6329625	0.097948	0.1252285	0.1332768	0.15925	0.25675	0.30025	0.19975
−9.066027	−40.33539	Fabricante, J.R., 2007 [19]	24		0.1845367	0.140596	0.2739415	0.222109	−0.158286	0.1571847	1.4529965	0.064957	0.1222073	0.1312205	0.14375	0.20875	0.28675	0.2245
−9.542444	−40.45658	Fabricante, J.R., 2007 [19]	33		0.4449937	0.3753287	1.43109175	1.311512	0.0730875	0.3768393	2.6335237	0.148949	0.1075	0.10436	0.09325	0.242	0.2105	0.1325
−6.616666	−37.28333	Silva, J.A., 2005 [2]	22	2.24	0.18371075	0.133216	0.27278775	0.21924	−0.2169892	0.148953	1.4507587	0.05155	0.1173468	0.1079155	0.11475	0.1665	0.2625	0.18
−6.976485	−37.58781	Silva, J.A., 2005 [2]	32	2.45	0.1950975	0.1439137	0.29614275	0.270992	−0.1603037	0.1675572	1.4912405	0.070233	0.1165768	0.1252743	0.1495	0.21975	0.304	0.2085
−9.058301	−40.3291	Calixto-Júnior & Drumond, 2014 [3]	16	1.39	0.23491	0.1778925	0.3515965	0.4143425	−0.1600405	0.197483	1.6142145	0.076167	0.116548	0.1070725	0.1240955	0.2002615	0.276581	0.59905
−14.283333	−44.45	Santos, R.M., 2006 [4]	19	2.94	0.26928175	0.1889845	0.4237435	0.556145	−0.095876	0.2186585	1.7375605	0.078454	0.1063085	0.096674	0.108173	0.186627	0.224512	0.13767
−7.271111	−37.27417	Leite, J.A.N., 2010 [5]	46	2.69	0.32024125	0.222882	0.588359	0.76438175	0.0942158	0.2693755	1.9478258	0.104255	0.1025368	0.106021	0.111	0.2155	0.2605	0.18025
−9.08	−40.319	Lima Júnior et al., 2014 [6]	5		0.27403	0.13168	0.44354925	0.57566	−0.16327	0.20764	1.7572163	0.06007	0.09654	0.085795	0.08075	0.141	0.19675	0.1405
−9.08	−40.321	Lima Júnior et al., 2014 [6]	5		0.25234	0.1575	0.3943165	0.48628	−0.15425	0.19441	1.676512	0.05944	0.0980603	0.0889688	0.0885	0.148	0.202	0.146
−9.079	−40.32	Lima Júnior et al., 2014 [6]	4		0.27168	0.1582	0.4364895	0.56609	−0.10537	0.20133	1.7470525	0.05609	0.09274	0.0818275	0.0752	0.131	0.1695	0.11325
−9.057	−40.329	Lima Júnior et al., 2014 [6]	5		0.29572	0.17404	0.49669175	0.66554	−0.11855	0.22094	1.8412315	0.06261	0.0912198	0.0802405	0.07475	0.13725	0.17425	0.1135
−9.07	−40.313	Lima Júnior et al., 2014 [6]	9		0.35271	0.20845	0.67643125	0.89382	−0.04526	0.26194	2.09398	0.07331	0.0885593	0.076273	0.06725	0.14075	0.15	0.09725
−9.069	−40.313	Lima Júnior et al., 2014 [6]	6		0.29399	0.17145	0.4912995	0.65849	−0.06659	0.21756	1.8335735	0.17145	0.0916	0.0802405	0.07275	0.133	0.152	0.104
−9.07	−40.312	Lima Júnior et al., 2014 [6]	3		0.3334	0.20279	0.61277225	0.81708	−0.04613	0.24912	2.00615	0.07081	0.09274	0.081034	0.07075	0.1415	0.15075	0.401
−9.058	−40.329	Lima Júnior et al., 2014 [6]	3		0.2596	0.1544	0.40928725	0.51607	−0.16423	0.19716	1.7022565	0.0581	0.09312	0.084208	0.0835	0.14125	0.1975	0.1385
−9.079	−40.342	Lima Júnior et al., 2014 [6]	2		0.19173	0.10904	0.28458575	0.24762	−0.224825	0.14448	1.4749672	0.04171	0.0988203	0.088969	0.088	0.1295	0.205	0.14325
−9.081	−40.32	Lima Júnior et al., 2014 [6]	4		0.277933	0.16874	0.4510845	0.59204	−0.11471	0.21048	1.788605	0.06144	0.09464	0.0850015	0.07975	0.14125	0.1785	0.1225
−9.058	−40.328	Lima Júnior et al., 2014 [6]	2		0.258659	0.14836	0.4071815	0.51212	−0.12648	0.19108	1.6987717	0.05291	0.0923598	0.081034	0.076	0.12875	0.166	0.111
−9.079	−40.32	Lima Júnior et al., 2014 [6]	3		0.27167	0.1582	0.4364895	0.56609	−0.10537	0.20133	1.322008	0.05599	0.09274	0.0818275	0.07525	0.131	0.1625	0.11325
−9.033	−40.315	Lima Júnior et al., 2014 [6]	2		0.237428	0.15034	0.368526	0.42761	−0.1279	0.183003	1.62802	0.05576	0.1014805	0.0924383	0.09075	0.14625	0.18925	0.1305
−9.081	−40.321	Lima Júnior et al., 2014 [6]	3		0.274609	0.16376	0.4434425	0.57818	−0.10023	0.206593	1.719503	0.05943	0.0935	0.0834145	0.07875	0.107625	0.1685	0.1165
−6.616666	−37.46667	Amorim et al., 2005 [7]	15	1.94	0.18859	0.12765	0.279291	0.23506	−0.21581	0.15452	1.464858	0.05506	0.107429	0.1021665	0.118	0.17375	0.26225	0.17625
−8.566667	−38.13333	Rodal et al., 2008 [8]	27		0.21189	0.15522	0.235489	0.31259	−0.21327	0.17778	1.3657672	0.06815	0.1249845	0.126953	0.142	0.193	0.324	0.25525
−8.3	−38.58333	Rodal et al., 2008 [8]	26		0.210498	0.1642345	0.274288	0.3183775	−0.189959	0.176945	1.534293	0.066308	0.1236035	0.1240695	0.13475	0.1995	0.3025	0.22725
−9.078408	−40.31897	Calixto-Júnior & Drumond, 2011 [9]	16	1.39	0.258858	0.16901	0.424352	0.545844	−0.097297	0.208136	1.7279975	0.065064	0.097084	0.089037	0.0895	0.15175	0.1895	0.12475
5.938548	−38.0567	Bessa & Medeiros, 2011 [10]	21		0.575632	0.4507135	2.27771875	1.6229185	0.237384	0.470681	3.716364	0.167119	0.089682	0.0869055	0.062	0.233	0.1545	0.08825
−7.471506	36.8963	Barbosa et al., 2007 [11]	12	2.25	0.168303	0.1042995	0.2467585	0.1665172	−0.2396	0.1313043	1.4048065	0.040875	0.104965	0.0955553	0.1015	0.1425	0.22975	0.15925
−7.396667	−36.53194	Barbosa et al., 2007 [11]	20	1.42	0.21202	0.173996	0.3169285	0.323396	−0.160678	0.18657	1.538501	0.08238	0.129212	0.138949	0.1575	0.2425	0.33525	0.25975
−9.065833	−40.33528	Fabricante et al., 2012 [12]	26	1.89	0.216505	0.13641	0.3242925	0.265901	−0.18129	0.16873	1.4898967	0.0581	0.1106013	0.1143598	0.11975	0.178	0.2565	0.20325
−9.542222	−40.45639	Fabricante et al., 2012 [12]	34	2.69	0.181086	0.12533	0.26883925	0.210081	−0.178678	0.1516	1.44276	0.058275	0.1099645	0.115689	0.13275	0.19125	0.274	0.18925
−8.311944	−38.19583	Rodal et al., 2008 [13]	28		0.27586	0.203976	0.839125	0.58345	−0.1361315	0.2308525	1.7622625	0.087725	0.109104	0.111095	0.1115	0.195	0.267	0.19325
−8.510218	−37.9853	Maragon *et* al., 2013 [14]	18	2.11	0.1391355	0.0911435	0.2145135	0.084943	−0.245212	0.1122285	1.323307	0.03826	0.1089175	0.108098	0.12	0.16225	0.2615	0.1985
−6.664722	−36.81778	Costa et al., 2009 [15]	31		0.17372	0.130547	0.260308	0.183888	−0.176519	0.14839	1.4254595	0.061004	0.124733	0.131032	0.14	0.20075	0.285	0.20925
−3.683333	−40.33333	Campanha et al., 2011 [16]	16	1.62	0.2543265	0.1594905	0.4028105	0.47447	−0.089749	0.1979415	1.6743113	0.060315	0.107048	0.102554	0.09775	0.16525	0.16325	0.11725
−8.238333	−35.92222	Alcoforado Filho et al., 2003 [17]	55	3.09	0.570529	0.472859	2.159367	1.608693	0.137581	0.479954	3.66649	0.185585	0.092364	0.089455	0.06675	0.2635	0.19125	0.106
−6.881111	−35.79472	Pereira et al., 2001 [18]	26		0.65799	0.5894	3.42972	1.826161	0.26139	0.56007	4.86472	0.225091	0.090626	0.080737	0.05925	0.28425	0.166	0.07725
−6.81	−36.96056	Fabricante et al., 2007 [19]	20	1.96	0.28229	0.219936	0.394627	0.610146	−0.07777	0.242746	1.187122	0.101107	0.113529	0.111722	0.12975	0.233	0.26175	0.1645
7.016667	−37.4	Guedes et al., 2012 [20]	21	2.54	0.200213	0.15427	0.3101125	0.277858	−0.219557	0.172277	1.500744	0.071922	0.121617	0.121563	0.135	0.20575	0.31825	0.2105
−6.881111	−35.795	Pereira et al., 2002 [21]	54	2.99	0.64377	0.566551	3.1764095	1.79771	0.199925	0.548299	4.61482	0.22084	0.089883	0.080737	0.05975	0.28275	0.166	0.08025
−7.52	−35.99972	Souza et al., 2007 [22]	36	2.64	0.260751	0.194672	0.410643	0.520689	−0.177221	0.219813	1.705477	0.08554	0.1114225	0.1117535	0.11875	0.20375	0.2865	0.214
−7.9025	−37.15194	Pegado et al., 2006 [23]	35	2.81	0.3002055	0.25744	0.501041	0.684229	−0.033717	0.2580802	1.8445762	0.119415	0.1254265	0.126134	0.1455	0.264	0.29025	0.2065
−7.893333	−37.14333	Pegado et al., 2006 [23]	16	0.61	0.255483	0.201091	0.4005395	0.499074	−0.10995	0.22228	1.687431	0.093599	0.1222648	0.124786	0.15	0.2375	0.2955	0.21725
−5.553889	−37.88861	Pessoa et al., 2008 [24]	8	1.1	0.14719575	0.0996635	0.22465475	0.1080907	−0.2565288	0.1218195	1.3534723	0.044838	0.110629	0.113717	0.1305	0.1755	0.296	0.22725
−5.537778	−37.89556	Pessoa et al., 2008 [24]	7	0.86	0.25523625	0.187931	0.4005857	0.4980805	−0.1575988	0.2125665	1.6870203	0.079582	0.1161188	0.1107465	0.116	0.19575	0.269	0.172
−7.3791	−36.5297	Araújo et al., 2010 [25]	14		0.208282	0.151041	0.3110365	0.3093737	−0.126106	0.1776147	1.526859	0.071545	0.111697	0.1161795	0.13675	0.20875	0.2655	0.19325
−6.854444	−41.47417	Mendes, M.R. A., 2003 [26]	33	2.96	0.2825747	0.192125	0.4614342	0.6113492	−0.0983102	0.2311432	1.787798	0.081976	0.099164	0.0934345	0.104	0.186	0.2265	0.15425
−4.805103	−38.75151	Barbosa et al., 2014 [27]	22		0.108927	0.089229	0.18255225	0.015626	−0.311471	0.089378	1.244542	0.032049	0.1401758	0.1298958	0.13075	0.16275	0.31	0.2485
−6.295	−39.33306	Braga & Cavalcante, 2007 [28]	21	2.67	0.337279	0.268802	0.618786	0.834229	0.0184	0.288918	2.018527	0.119791	0.110765	0.1106855	0.1175	0.234	0.2315	0.13975
−8.8	−39.83333	Drumond et al., 1982 [29]	26		0.241658	0.161878	0.3781472	0.4438437	0.108724	0.192775	1.6436207	0.063873	0.1033805	0.0923978	0.0995	0.1635	0.20525	0.13625
−8.15	−36.32083	Andrade et al., 2009 [30]	32		0.591008	0.475002	2.22555825	1.662462	0.227707	0.5103605	3.902935	0.216279	0.0871575	0.09181	0.082	0.2735	0.1925	0.10425
−5.356666	−39.41778	Mourão, A.E.B., 2013 [31]	11	0.98	0.28297	0.22028	0.46821925	0.62255475	−0.19825	0.24441	1.79413	0.104035	0.1133218	0.1086415	0.133	0.23725	0.3545	0.6125
−96652777	−37.66944	Ferraz et al., 2013 [32]	24		0.38649	0.32042	0.19354975	0.056215	−0.01779	0.3333	2.26439	0.140438	0.109787	0.11395	0.111	0.25225	0.2585	0.17825
−9.081	−40.32	Lima Júnior et al., 2014 [6]	8		0.27793	0.16874	0.4510845	0.59205	−0.08335	0.21048	1.7706648	0.06144	0.09464	0.0850015	0.0795	0.14125	0.1785	0.1225
−9.069	−40.312	Lima Júnior et al., 2014 [6]	9		0.28552	0.1666	0.470932	0.62336	−0.1112	0.21037	1.8010867	0.0579	0.09236	0.0818275	0.0725	0.13025	0.1635	0.105
−9.08	−40.32	Lima Júnior et al., 2014 [6]	7		0.21863	0.13987	0.34331425	0.34924	−0.19715	0.17234	1.55998	0.05542	0.101101	0.0984903	0.0992	0.1545	0.231	0.1765
−9.032	−40.314	Lima Júnior et al., 2014 [6]	4		0.24483	0.15273	0.37759275	0.45502	−0.1444	0.18799	1.6488145	0.05659	0.0988203	0.088969	0.0872	0.144	0.1925	0.4207
−9.079	−40.32	Lima Júnior et al., 2014 [6]	7		0.27168	0.1582	0.4364895	0.56609	−0.10537	0.20133	1.7470525	0.05609	0.09274	0.0818275	0.0752	0.131	0.1695	0.11325
−10	−40.315	Lima Júnior et al., 2014 [6]	2		0.27881	0.17727	0.4529515	0.59569	−0.1128	0.217	1.773831	0.06746	0.09692	0.0913495	0.08725	0.15475	0.19425	0.12975
**MEAN**					0,27929116	0,19924978	0,59623114	0,56678483	−0,099698	0,2268671	1,8694487	0,0836	0,1058698	0,1018481	0,1050178	0,1857919	0,2313516	0,18236
**STANDARD DEVIATION**					0,11600442	0,10765524	0,66934308	0,41329868	0,1243024	0,0999113	0,7485234	0,045136	0,012777	0,0174945	0,0281493	0,0462248	0,0562699	0,10236.

Note: R-richness; S Shannon's index; Vegetation Index - NDVI: Normalized Difference Vegetation Index, EVI:Enhanced Vegetation Index, LAI:Leaf area index, NDWI: Normalized Difference Moisture Index or Water Index, SAVI: Soil-Adjusted Vegetation Index,SR: Simple Ratio Index, DVI: Difference Vegetation Index. Spectral band – BLUE, RED, GREEN, NIR: Near-infrared, SWIR: Short-wavelength infrared.

## 2 Experimental design, materials and methods

### 2.1 Data on richness and Shannon's index

Phytosociological studies were carried out in Caatinga. Sixty richness data and twenty-five Shannon indices were extracted and used in this research (Table 1).

### 2.2 Image acquisition

Thematic Mapper (TM) and Operational Land Imager (OLI) images from the Landsat 5 and 8 satellites were used. These images were acquired from the Global Visualization Viewer (GloVis) of the US Geological Survey (USGS). The methods to extract vegetation indices and spectral bands were described in the research article “Predicting plant species richness with satellite images in the largest dry forest nucleus in South America”.

### 2.3 Leaf-level spectral reflectance

The leaf spectral reflectance (Supplementary Material 1 – SM1) between 336 and 1045 nm with a resolution of 1 nm was measured using a spectroradiometer (model FieldSpec® HandHeld Pro) and it used a 1 and 10° HH FOV lens foreoptic with radiometric calibration. The measurements were taken in a pristine caatinga area around 9°2′47.62″S and 40°19′16.67″W. It was selected eight representative species: 1- *Manihot glaziovii*; 2- *Croton sonderianus*; 3- *Jatropha mollissima*; 4- *Croton conduplicatus*; 5- *Commiphora leptophloeos*; 6- *Bauhinia* sp., 7- *Capparis flexuosa* L., and 8- *Cereus jamacaru*. The leaf level reflectance variation from eight plant species was presented in box-plot graphics, with median, maximum, minimum and quartz values (Fig. 1).
